# Supplementary material for: Palaeogenomic analysis of black rat (Rattus rattus) reveals multiple European introductions associated with human economic history
Source: Nat Commun. 2022 May 3;13:2399. doi: 10.1038/s41467-022-30009-z (PMC9064997; doi:10.1038/s41467-022-30009-z)
Supplement: Supplementary file 3 — Description of Additional Supplementary Files [file 41467_2022_30009_MOESM3_ESM.pdf]

Description of Additional Supplementary Files

File name: Supplementary Data 1.

Description: The summary statistics of G-PhoCS, including:

Data S1.1 The summary of statistics from the GPhoCS preliminary run

Data S1.2 The parameter priors and summary statistics from the final GPhoCS runs

File name: Supplementary Data 2.

Description: The metadata for rat samples involved in different types of analysis, including:

Data S2.1 The information of all black rat samples screened for next generation sequencing

Data S2.2 The samples included in mitochondrial genome analysis and their haplotype information

Data S2.3 Information of samples with newly generated CYTB fragments

Data S2.4 Information for all the newly reported and published samples included in mitochondrial fragment analysis

Data S2.5 Sample information and NGS statistics of rat samples included in nuclear genome analysis

File name: Supplementary Data 3.

Description: Radiocarbon dates on samples included in whole genome analysis

File name: Supplementary Data 4.

Description: F_4-statistics based on autosomal genotypes, including:

Data S4.1. F_4-statistics for the grouping of samples from the same site.

Data S4.2. F_4-statistics in the form of f_4(Outgroup, Med; Roman1, Roman2) to test the affinity of medieval populations to different Roman populations.

Data S4.3. F_4-statistics in the form of f_4(Outgroup, Roman; Med1, Med2) to test the affinity of Roman populations to different medieval populations.

Data S4.4. F_4-statistics to test the affinity between two Buda Castle populations and the other medieval populations from temperate Europe

File name: Supplementary Data 5.

Description: Coverage summary on Y-chromosome scaffolds for selecting scpMSY regions.

Source Data: Source data for the main and supplementary figures are included in Supplementary Data 1 and 4.
