# Supplementary material for: Palaeogenomic analysis of black rat (Rattus rattus) reveals multiple European introductions associated with human economic history
Source: Nat Commun. 2022 May 3;13:2399. doi: 10.1038/s41467-022-30009-z (PMC9064997; doi:10.1038/s41467-022-30009-z)
Supplement: Supplementary file 9 — Reporting Summary [file 41467_2022_30009_MOESM9_ESM.pdf]

nature portfolio

Corresponding author(s): Johannes Krause
Greger Larson
David Orton

Last updated by author(s): Feb 7, 2022

Reporting Summary

Nature Portfolio wishes to improve the reproducibility of the work that we publish. This form provides structure for consistency and transparency in reporting. For further information on Nature Portfolio policies, see our Editorial Policies and the Editorial Policy Checklist.

Statistics

For all statistical analyses, confirm that the following items are present in the figure legend, table legend, main text, or Methods section.

n/a	Confirmed	
☐	☒	The exact sample size (n) for each experimental group/condition, given as a discrete number and unit of measurement
☐	☒	A statement on whether measurements were taken from distinct samples or whether the same sample was measured repeatedly
☒	☐	The statistical test(s) used AND whether they are one- or two-sided<br>*Only common tests should be described solely by name; describe more complex techniques in the Methods section.*
☒	☐	A description of all covariates tested
☐	☒	A description of any assumptions or corrections, such as tests of normality and adjustment for multiple comparisons
☐	☒	A full description of the statistical parameters including central tendency (e.g. means) or other basic estimates (e.g. regression coefficient) AND variation (e.g. standard deviation) or associated estimates of uncertainty (e.g. confidence intervals)
☒	☐	For null hypothesis testing, the test statistic (e.g. F, t, r) with confidence intervals, effect sizes, degrees of freedom and P value noted<br>*Give P values as exact values whenever suitable.*
☐	☒	For Bayesian analysis, information on the choice of priors and Markov chain Monte Carlo settings
☒	☐	For hierarchical and complex designs, identification of the appropriate level for tests and full reporting of outcomes
☒	☐	Estimates of effect sizes (e.g. Cohen's d, Pearson's r), indicating how they were calculated

Our web collection on statistics for biologists contains articles on many of the points above.

Software and code

Policy information about availability of computer code

Data collection	Meraculous 2.2.5.1<br>HiRise scaffolding pipeline<br>Geneious R6 version 6.0.6<br>EAGER 1.92.55<br>AdapterRemoval 2.2.0<br>BWA 0.7.12<br>DeDup 0.12.1<br>mapDamage 2.0.6<br>bamUtil 1.0.13<br>GATK v3.5<br>CircularMapper 1.0<br>Schmutzi 1.0<br>ANGSD 0.931<br>SAMtools 1.3<br>BCFtools 1.3<br>vcftools 0.1.15<br>pileupCaller 1.2.2<br>MUSCLE v3.8.1551<br>MAFFT v7.123b
Data analysis	OxCal 4.4<br>RepeatMasker 4.0.7

TRF 4.09
BUSCO 3.0.2
nucmer 4.0.0
PSMC 0.6.5
G-PhoCS 1.2.3
jmodeltest v2.1.10
RAxML v8.2.12 and v8.2.9
Ape 5.3
R 3.5.1
PLINK v1.90b
ADMIXTOOLS 5.1
Treemix 1.13

For manuscripts utilizing custom algorithms or software that are central to the research but not yet described in published literature, software must be made available to editors and reviewers. We strongly encourage code deposition in a community repository (e.g. GitHub). See the Nature Portfolio guidelines for submitting code & software for further information.

Data

Policy information about availability of data

All manuscripts must include a data availability statement. This statement should provide the following information, where applicable:
- Accession codes, unique identifiers, or web links for publicly available datasets
- A description of any restrictions on data availability
- For clinical datasets or third party data, please ensure that the statement adheres to our policy

The black rat genome assembly is available in the NCBI under the accession number GCA_011800105.1 [https://www.ncbi.nlm.nih.gov/assembly/GCA_011800105.1/]. Aligned reads from the 39 newly reported ancient and modern black rats are available at the ENA archive under the accession number PRJEB47337 [https://www.ebi.ac.uk/ena/browser/view/PRJEB47337]. The mitochondrial genome haplotypes are available at NCBI GeneBank under the accession number OK210796 - OK210933.

Field-specific reporting

Please select the one below that is the best fit for your research. If you are not sure, read the appropriate sections before making your selection.

☒ Life sciences ☐ Behavioural & social sciences ☐ Ecological, evolutionary & environmental sciences

For a reference copy of the document with all sections, see nature.com/documents/nr-reporting-summary-flat.pdf

Life sciences study design

All studies must disclose on these points even when the disclosure is negative.

Sample size	The number of analyzed samples is determined based on their endogenous DNA content and sampling locations. For the sampling sites with a lot of high-quality samples, we only selected at most four samples per site, to avoid an over-representation of any site in our final dataset.
Data exclusions	For mitochondrial DNA analysis: sequences with <90% sites covered are excluded from the analysis.<br>For whole genome analysis: sequencing reads with mapping quality/base quality<30 are excluded from genotyping
Replication	Replication is not applicable for ancient specimens as the specimen's weight is not enough for multiple sampling.
Randomization	Samples are grouped based on sampling sites and dates, and further tested using F-statistics.
Blinding	Blinding is not relevant in this study as the specimens were sampled based on their geographic locations and ages. Blinding in downstream analysis is not relevant either as we grouped them based on known locations and ages, and analyzed the genome-wide data in relation to other groups and publicly available data from Rattus rattus and its closely related species.

Reporting for specific materials, systems and methods

We require information from authors about some types of materials, experimental systems and methods used in many studies. Here, indicate whether each material, system or method listed is relevant to your study. If you are not sure if a list item applies to your research, read the appropriate section before selecting a response.

Materials & experimental systems

n/a	Involved in the study	
☒	☐	Antibodies
☒	☐	Eukaryotic cell lines
☐	☒	Palaeontology and archaeology
☐	☒	Animals and other organisms
☒	☐	Human research participants
☒	☐	Clinical data
☒	☐	Dual use research of concern

Methods

n/a	Involved in the study	
☒	☐	ChIP-seq
☒	☐	Flow cytometry
☒	☐	MRI-based neuroimaging

Palaeontology and Archaeology

Specimen provenance	Specimen provenance is described in Supplementary Note 3 and Data S2.1. In most cases, permits were not required for analysis of the ancient specimens, which were provided by co-authors on the paper who either excavated or already had access to the material. Specimens from Assos (Ass_1, Ass_2) were exported with the permission of the Çanakkale Museum, Turkey (77366169-152/766). Specimens from Rirha (AJ410-AJ414) were exported under a permit from the Ministry of Culture of Morocco. Rat bones from Panga Ya Saidi (AJ37-AJ38) and Chombo (AJ40-AJ41) were obtained and exported with permission from the National Council for Science and Technology, Kenya (Research Clearance Permit NCST/RRI/12/1/SS/541; Exploration/Excavation Licence NMK/GVT/2) and National Museums Kenya (export permit dated 12/09/2011). The sample from Songo Mnara (AJ40) was exported under permit EA.402/605/01/9 issued by the Antiquities Division, Ministry of Natural Resources and Tourism, Tanzania. Formal loan/sampling agreements were signed with: University of Tartu Archaeological Collection (samples AJ363, AJ366; Sampling Protocol #89), South Holland Provincial Archaeological Depot (samples AJ469-AJ472; loan number 2018-27), Åland Museum (samples AJ404-AJ409, ÅLR 2018/3788).
Specimen deposition	Curating institutions for sampled specimens/their parent collections are listed in Supplementary Data S2.1
Dating methods	New dates are generated from 14 specimens, as described in Method and Data S3, at Manheim (MAMS), University of Waikato (Wk) and Oxford University (OxA). The radiocarbon dates are calibrated in OxCal 4.4, using the IntCal20 calibration curve.

☒ Tick this box to confirm that the raw and calibrated dates are available in the paper or in Supplementary Information.

Ethics oversight	No ethical approval was required for ancient rat study.

Note that full information on the approval of the study protocol must also be provided in the manuscript.

Animals and other organisms

Policy information about studies involving animals; ARRIVE guidelines recommended for reporting animal research

Laboratory animals	No laboratory animal is used in this study.
Wild animals	A male, adult black rat (Rattus rattus) individual of reproductive age was wild-trapped to get the fresh tissue for high-quality genome sequencing. The animal was captured alive by the Alameda County Vector Control District in a large wire mesh trap, then transported in the trap in a truck to UC Berkeley. We used the Isoflurane drop technique to euthanize it. The individual was made into specimen and cataloged at the Museum of Vertebrate Zoology, UC Berkeley, https://arctos.database.museum/guid/MVZ:Mamm:236302.
Field-collected samples	No field-collected animal is used in this study.
Ethics oversight	No ethical approval was required for capture of the black rat.

Note that full information on the approval of the study protocol must also be provided in the manuscript.
